# Hospital and Institutional News

**Published:** 1916-06-24

**Authors:** 


### HOSPITAL AND INSTITUTIONAL NEWS.

### THE COUNCIL ELECTION OF THE ROYAL COLLEGE OF SURGEONS.

For the four vacancies on the Council of the Royal College of Surgeons there are seven competitors, only one of whom is standing for re-election: this is Mr. W. F. Haslam, of Birmingham, and as he is the only provincial candidate, he should be fairly certain of retaining his seat. The others are Mr. T. H. Openshaw, C.M.G.; Mr. Raymond Johnson; Mr. John Murray; Mr. V. W. Low, C.B.; Mr. H. S. Pendlebury; and Mr. F. J. Steward. On the whole the most striking feature is the comparative youth of this list. Mr. Haslam is the senior, but of the newcomers there is none whose fellowship dates back more than thirty years, and two who are of less than twenty years' standing in that respect. This is as it should be; a proportion of men still no more than middle-aged is highly beneficial to a Council such as this, where of necessity many of the members are past their prime. There should be a fairly close contest amongst these candidates; so much so that, beyond the "selection" already mentioned, we should be very loth to predict which four will secure election and which will be the three who will not do so. The ballot is on July 6.

### INSANITY AND THE SERVICES.

Dr. Shaw, medical superintendent of the Montrose Royal Infirmary, records the result of his experience as to the degree in which the war has affected the asylum population. In his report he has been able to state that the effect has been extremely small, while the two patients who had had experience of active service had made good recoveries. Of two admitted during the past year from Service battalions, one had suffered from a mental breakdown in the past. The other, congenitally deficient, had been only a few weeks in the Army. It would be interesting to know how he came to be admitted at all, and who was the medical officer responsible for passing him. Again, of two men in the Naval Reserve one was a mine-sweeper, unused previously to a life afloat, who suffered from depression, as one can well believe, from so dangerous, lonely, and monotonous a task. Physical fitness and mental stability are closely connected; that is why it is so important for the medical examination of recruits to be thorough.

### ANOTHER MEDICAL CONSCIENTIOUS OBJECTOR.

Mr. John Cameron MacCallum, M.B., Ch.B., lately executive tuberculosis officer and assistant medical officer for the County of Argyll, has been fined £2 for failing to report himself at Stirling on the prescribed date, in accordance with the requirements of the Military Service Act, notice of which had been served on him. While admitting a breach of the statute, Dr. MacCallum pleaded "Not guilty" on the ground that his conscientious objections to participating in war in any capacity made it impossible for him to comply. He had applied for a certificate of exemption as a conscientious objector, but it had not been received by him. Dr. MacCallum left the dock in the custody of a military escort.

### KING EDWARD VII.'S HOSPITAL, CARDIFF.

Reference was made at a recent meeting of the board of management of King Edward VII.'s Hospital, Cardiff, to recent generous gifts which have greatly enhanced the usefulness of the hospital. Sir Edward Nicholl's donation of £10,000 is enabling the board to build a new flat accommodating thirty-two beds. As was mentioned in our columns last week, the generosity of Mr. and Mrs. W. H. Seager makes possible the immediate building and equipment of a new operating theatre. The tender of Messrs. Knox and Wells has been accepted for the extensions to the massage department, and tenders for the new children's ward and the maternity flat are being invited. Colonel Bruce Vaughan, in commenting upon these benefactions, said they would make the addition of a further 150 beds to the hospital quite possible in the near future. In normal times the hospital, with its 300 beds, would treat about 3,000 in-patients per annum, but these additions would enable 1,500 more in-patients to be treated. The next item in the hospital's programme is the raising of an endowment fund of £100,000 for the provision of research scholarships, fellowships, medical and surgical scholarships, and prizes for medical students. Thus there is nothing lacking in ambition or enterprise on the part of those charged with the management of this institution.

### MORE ZEPPELIN HOSPITAL FINANCE.

In the income and expenditure of a certain hospital the revenue from collection-boxes is shown as nearly £100 more than in the previous year. This variation would hardly elicit remark; but the receipts from boxes rarely change much in volume, for this means of raising funds is not much in favour with most secretaries and is retained more for advertisement than direct profit. With all regard to dutiful reticence it may be admitted that the phenomenon in question was entirely due to a Zeppelin raid, and these are the facts: the German visitors on the way to some dockyards many miles away accidentally dropped part of their cargo in a quiet residential district where there happens to be a hospital, the windows of which were as a result in most cases blown out. Enormous crowds visited the spot next day, and the secretary, knowing that the Censor would not allow newspaper publicity, was perplexed as to what steps to take to make the best of a bad business. He decided to attempt to awaken practical sympathy in the crowd and posted a notice on the front porch stating that nobody had been hurt, but that the hospital had been hit in more ways than one during the war and that contributions in the adjacent box would be thankfully received. The combination of this appeal and the dishevelled appearance of the building elicited gifts to the extent of £150 in less than a week; two-thirds of this sum was given through the agency of the collecting-box.

### THE GREATEST MODERN EPIC.

By the time that this issue is in the hands of our readers the British and Russian Red Cross Societies will have benefited by the proceeds of a remarkable performance which was held at Weymouth on Thursday. It consists of a series of scenes, presented by the Dorchester Players, from "The Dynasts" of Mr. Thomas Hardy. All discriminating persons attended the representations given in London, shortly after the outbreak of war, of scenes from this epic drama, and noted the irony of a situation in which the one great ready-made topical play of that alarming moment was of all things an epic: the one form of art which is popularly presumed to be wholly dead. At Weymouth the scenes selected from the nineteen acts of this great play are those which depict the scares of invasion which overran Wessex during the dazzling period of Bonaparte's success.

### THE LATEST ANTI-SYPHILITIC REMEDIES.

A brief but very succinct and business-like account has been published by Lieut.-Colonel Harrison, D.S.O., and Temporary Captain C. H. Mills, R.A.M.C., of their experience with two of the most recent remedies for syphilis, which have been greatly eulogised by Mr. J. E. R. MacDonagh. As a result of the latter's views upon the biology of the spirochœte, or causal micro-organism, of syphilis, and of his theory of its chemical reactions and vital processes, he was led to a hypothesis that a certain compound of sulphur, intramine, and a certain compound of iron, ferrivine, would be just as efficacious in the treatment of syphilis as the poisonous arsenical compounds introduced a few years ago by Ehrlich. This hypothesis he has put to the test of experiment, and is very well satisfied with the results. Institutional medical officers who are thinking of trying these new drugs on their patients will do well to heed first of all the warning notes sounded by Colonel Harrison. His conclusions, which appear to be quite justified by his experiences with these two drugs, are most unfavourable. They are that intramine and ferrivine are extremely unpleasant in their effects; and that they have no specific effect on early syphilis. Further, it would seem as if the use of both presents very real and serious danger to the patient. It is not likely that Mr. MacDonagh will be content to leave it at that; but Colonel Harrison has such a deservedly high reputation as an expert on syphilis as well as for common-sense and honesty that the former may find the convincing of the profession no easy task.

### ADVERTISEMENTS IN VOLUNTARY HOSPITALS.

In the columns of our contemporary, *Education*, a controversy has been started between a London consulting physician and a firm which manufactures condensed milk, with regard to the value of the latter product as a foodstuff for infants and young children. Into the merits of this public discussion we do not propose to enter; but a curious revelation has been made in the course of it which raises a point for managers of voluntary hospitals. It is stated that recently the firm in question (one of very good repute) offered to a London hospital which was asking for donations the sum of £400 to name a cot after the condensed milk brand, on condition that the hospital should investigate any fifty cases of infant feeding in 1915 from the records kept by the firm. The offer was declined, presumably because the hospital was averse from appearing to accept endowments which were tantamount to a perpetual advertisement of one particular make of infant dietary. If that was the motive of the non-acceptance, we think the managers of the hospital did rightly; obviously it would be very difficult to draw the line if once the principle were granted that the walls of a hospital ward might be turned into an advertisement hoarding. Again, the mere fact that a particular firm is well conducted and of a good reputation is no absolute guarantee that it will always remain so; whereas a cot or bed once endowed, and the fact commemorated by an inscription in the usual way, must remain so in perpetuity, or at least could only be altered at great trouble and inconvenience.

### THE SPORTSMEN'S AMBULANCE FUND.

The committee, of which Lord Lonsdale is the active President, for raising a fund of £50,000 to provide the Allied Powers with a hundred motor ambulances as a gift from British sportsmen, is casting a wide net and displaying great energy. Over £3,000 is already to hand, and the committee are sufficiently optimistic of success that they have ordered a first instalment of fifty cars, to be delivered in a fortnight for presentation to our Allies. Two cars were placed on exhibition at a race meeting lately, and collections were made on the course by well-known actors and actresses; this example is to be followed at all future race meetings while the fund remains open. Many boxing promoters and managers are helping the committee, and a boxing carnival in aid of the fund is to be held at the National Sporting Club. The billiard professionals, through their Control Club, are about to assist in a similar way at a benefit tournament, and extensive arrangements for collections in theatres and music-halls are also on the programme. It should not be long before the committee are in a position to order the other fifty ambulances, considering the care with which the organisation is being carried out.

### TWO RICHMOND WAR HOSPITALS.

The old Richmond Horse Show has been transformed this year into the Richmond Red Cross Royal Horse Show, and the whole of the proceeds of it are to be devoted to Queen Mary's Star and Garter Home for totally disabled sailors and soldiers. Several new features, including a "costers' Marathon," were introduced into the show, which was favoured with a fine day and was attended by the Queen, Queen Alexandra, and King Manuel's Consort, and the Grand Duchess George of Russia. This is only one out of several novel methods which have been adopted in the last few weeks for raising money on behalf of the Star and Garter Home, close to which, inside the gates of Richmond Park, is situated the South African Military War Hospital, recently handed over to the War Office upon its completion. This hospital contains 304 beds, and its entire cost has been defrayed by the South African community in this country. It is in charge of Colonel Stock and is staffed by South African nurses. The site was granted by the King, and the buildings and equipment are worthy of the situation of the hospital in the Royal Park. Mr. Allison is the architect and Mr. McFerran the engineer responsible for the construction; both of these gentlemen gave their services.

### THE ROYAL LIVERPOOL COUNTRY HOSPITAL, HESWALL.

At the annual meeting of the governors of this institution, presided over by the Lord Mayor of Liverpool, allusion was made to the fact that it is the fourth largest of the voluntary charities of that city. It is doubtless owing to the hospital being outside Liverpool that it is not quite so much in the public eye there as some of the smaller institutions are; and the same fact militates to some extent, no doubt, against the financial support which it receives. During the past year the average number of beds occupied was 144; owing to the long duration of the average stay of each patient (nearly six months) the number of children passing through the hospital in one year is comparatively small (432). An arrangement has been made whereby only non-pulmonary or "surgical" tuberculosis cases are admitted; for pulmonary cases the Liverpool Insurance Committee now undertakes the responsibility. The special school at the hospital, whereby about three-fourths of the patients are given education, some of them for the first time in their lives, has earned a very good report from the inspectors of the Board of Education. The Lord Mayor expressed the hope that it would soon be possible to build the extra wards which the committee have in view; but we note that, in spite of a fairly successful year, the institution is still £1,500 overdrawn at the bank. During the year 142 soldiers were treated, at the expense of Mr. Herdman, in two small wards given up for that purpose.

### INQUESTS IN WAR TIME.

One of the coroners for the county of Durham, Mr. John Graham, has for twenty years adopted a course of procedure with regard to natural deaths which goes far to meet the suggestions for labour saving in connection with Coroners' juries made lately in the Press and commented on in The Hospital, June 17, p. 253. Mr. Graham, with the concurrence of the County Council, and his colleagues in the county are in the habit of paying a "moderate fee" to medical men for reliable written reports respecting all deaths from (presumably) natural causes; and thousands of such cases have been so dealt with "safely and without any complaint." In fact, from figures given by this Coroner it appears that in only about 40 per cent. of the cases reported to him does he hold an inquest: the tremendous saving of the time (and therefore of the labour and the money) of those on his jury rolls needs no explanation. We cannot help wondering whether the failure of this procedure to "catch on" all over the kingdom may not be due to the practice of remunerating the Coroner by a fee for each inquest held. Many local authorities have already substituted for this practice a fixed salary, and in our judgment this example should be followed everywhere.

### A YOUTHFUL "GAZETTE."

We are glad to note the adoption of our hint on the value of headlines in the second number of the *Gazette* of the 4th Southern General Hospital, Plymouth. A glimpse is given in this number of the work and time employed by Captain Smith and Sergeant-Major Griffiths, who have been busy providing a cinematograph entertainment for the men. There is also an article by "One of the Staff" on the achievements of the matron, Miss McKay, to the care of whose office, by the way, communications intended for the editor have to be addressed. A new departure is promised in the publication of a brief outline of the careers of the "honoured members" of the Territorial Force Nursing Staff, which could be made amusing as well as interesting. Such sketches are generally but a directory paragraph without the abbreviations, but since, in the nature of things, a long series of public distinctions cannot be recorded, mere human matters, such as the receipt of the bad conduct prize at school, or the awakening of interest in nursing through boredom with home life, should be inserted. Our careers begin with birth. It is largely circumstances which introduce us to hospitals. The *Gazette* at present requires a little more "body," which can best be provided by digging ino the actual working of each department (whose results alone are usually known), and into such facts about the district or patients' experiences as would be eagerly read.

### THE COLLECTORS OF HERBS.

In view of the question of the practicability of cultivating medicinal herbs in larger quantities in England and of using the gardens attached to the hospitals for this purpose, the following remarks on the collection of grass roots are of interest. They appeared in the *East Anglian Daily Times* in a letter signed John Rasor: "With regard to use of for medicinal purposes the roots of couch grass, spear grass, or twitch—for they all mean the same thing—as I have no doubt the farmers would only be too glad to be rid of it, it may be of interest to some of your readers to know how this may be done. For twitch, which may be gathered in quantity easily from the harrows after the harrowing of a ploughed field, the price offered is a shilling for every 25 lb., and cost of carriage is paid by party to whom sent. The same price is allowed for dandelion roots, but this depends on their age; first year roots are generally too small, and preference is given to second, third, or fourth year roots. I am informed that good collectors can average 25s. per week at this alone, where the roots are readily obtainable. A larger price is given for roots and tops of deadly nightshade." The occupation of collector here described is presumably a restricted one, but all facts are of interest which lead us to see how to develop Home resources.

### THE WAGES OF A HOSPITAL LABOURER.

Additional exemption under the Military Service Act was given last week at the Southwark Tribunal to a man nearly reaching the age limit who described himself as a "garden labourer and pony man" employed at Bethlem Hospital. In the course of examination the applicant remarked that for thirteen years his wages had been one pound a week with no extras, but that recently he had been receiving twenty-five shillings. Members of the Tribunal showed their surprise at the low wages which the man had been receiving, and eventually he was referred to the borough surveyor with a view to his getting better-paid work. It was suggested that he might well be employed as a caretaker in one of the open spaces controlled by the borough. Hospitals claim support for the standard which they set to the district wherein they are situated, and they should be models not only of kindness and skill to their patients, but model employers as well. To give a man in London such a small wage over so long a period is regrettable. On the score of faithful service alone twenty-five shillings a week, at war prices, in London is well below adequate remuneration for this work.

### A HOME FOR DEFECTIVES AT SHEFFIELD.

The Sheffield City Council has adopted a scheme for providing accommodation for sixty mental defectives at Wales Court, about ten miles outside the city. This property comprises a house of about fifty rooms, surrounded by a fine garden and an extensive orchard and seventy-nine acres of farmland. The whole estate has been secured on a "seven—fourteen—twenty-one" years' lease at a rental of £207 10s., which sounds extraordinarily cheap, especially as it is proposed to continue to let off the agricultural land for something over £100 a year. An estimate has been submitted for repair and adaptation of the premises amounting to £2,260, and it is said that for this sum Wales Court can be fully equipped for sixty patients. The house and grounds, so far as can be judged by mere description, sound as if they were ideal for the purpose which the Sheffield City Council has in view. The future work and development of this institution should be well worth watching.

### A SANATORIUM PROJECT ABANDONED.

At the annual meeting of the Royal National Sanatorium for Consumption and Diseases of the Chest, Bournemouth, which was held in London recently, it was reported that the committee which was appointed to consider a proposal for the establishment of a "sanatorium farm or colony" had turned its attention to a property situated near Bournemouth and formerly used as a sanatorium; but that the architects reported unfavourably, and the idea of purchasing the property was therefore abandoned. Whether the farm or colony which the managers had in mind was destined for the reception of patients in whom tuberculous disease had already been arrested by treatment in the institution itself, or was intended as a sanatorium for cases not yet completely arrested: in either case the desirability of securing the best possible conditions of site, climate, and construction is equally great, and a noteworthy shortcoming in any of these respects was quite properly to be regarded as a complete disqualification. We congratulate the National Sanatorium on its determination to do the best, and nothing but the best, for its patients.

### BRISTOL'S NEW CHILD-WELFARE SCHEME.

Members of the Health Committee and various representatives of voluntary organisations have united to form a Maternity and Child-welfare Committee in Bristol. In presiding at this opening meeting, Dr. Colston Wintle, chairman of the Health Committee, recalled that there were fourteen Schools for Mothers in Bristol, and that a superintendent and six other trained nurses had been appointed as health visitors. Miss Townsend, who represented the Council of the Schools for Mothers, also declared that over 1,000 mothers were now attending schools, where children also were being supervised, and, in spite of a variety of organisations, she did not think that there had been any overlapping. One of the chief aims of the new committee is to reduce the toll of death from measles and diarrhœa, and the new committee is widely representative of existing agencies.

### THIS WEEK'S DRUG MARKET.

Price changes of especial importance have not been numerous, and business has been rather quiet. Salicylic acid and salicylate of soda are still inclined downwards in price, and the general impression seems to be that values have not reached the lowest limits. The prices of bromides are by no means firmly maintained. Barbitone is very scarce and dearer. An extremely high figure has been paid for a small quantity of resorcin, and an equally high figure would no doubt have to be paid for anything further available. Sugar of milk continues to advance in value. Oil of cloves is dearer. There is little change in the position of tartaric acid and citric acid, but it is not improbable that prices will again recede. Dandelion root is much wanted, but there is very little to be had even at phenomenal rates. Herb growers in the Home Counties report that the lavender crop promises to be good. Belladonna and henbane are being cut earlier this year—for the purpose of making extracts—owing to the necessity of supplying the demand. Cod-liver oil is obtainable at lower rates, but the price is still far too high to tempt buyers.

